# Safeguarding equitable HIV service delivery at the health facility-level in a resource-limited setting during the pandemic

**DOI:** 10.1186/s41182-022-00441-4

**Published:** 2022-07-22

**Authors:** Rosario Jessica Tactacan-Abrenica, Dennis Gregg Almonte, Kristal An Agrupis, Greco Mark Malijan, Shuichi Suzuki, Ralph dela Cruz, Rich King Valdez, Lalaine Arcangel, Koya Ariyoshi, Chris Smith

**Affiliations:** 1 HIV and AIDS Department, San Lazaro Hospital, Manila, Philippines; 2 School of Tropical Medicine and Global Health, Nagasaki University, Nagasaki, Japan; 3 San Lazaro Hospital-Nagasaki University Collaborative Research Office, Manila, Philippines; 4 Institute of Tropical Medicine, Nagasaki University, Nagasaki, Japan; 5 Department of Clinical Research, London School of Hygiene and Tropical Medicine, London, UK

**Keywords:** HIV, PLHIV, COVID-19, LMIC, Resource-limited setting, Health service delivery

## Abstract

The COVID-19 pandemic had a severe impact on delivering essential health services, including HIV service delivery. Among the challenges encountered and addressed by the HIV and AIDS Department of the San Lazaro Hospital were ensuring continued access to antiretroviral therapy and ensuring continuity of client education and empowerment. Two years into the pandemic, challenges still ensue, such as protecting health care providers from COVID-19 and regular clinical monitoring of clients. This highlights the importance of urgent action to strengthen the resilience of health systems at all its levels, not only to respond to sudden disturbances, but also to transform and evolve to be able to better face future pandemics.

To the editor,

The HIV and AIDS Department of the San Lazaro Hospital (SLH-H4) is a major government-run treatment hub for people living with HIV (PLHIV) in the Philippines. Situated within the national referral hospital for infectious diseases, its outpatient service is responsible for the enrollment of new clients, follow-up check-ups of existing clients including antiretroviral therapy (ART) refills, and provision of pre- and post-test counselling services throughout the spectrum of HIV care. The operations team is composed of 3 doctors, 8 case managers, and 12 nurses. In 2019, H4 OPD provided 19,825 consultations including enrollment of 250 new clients (Fig. 1).

**Fig. 1:**
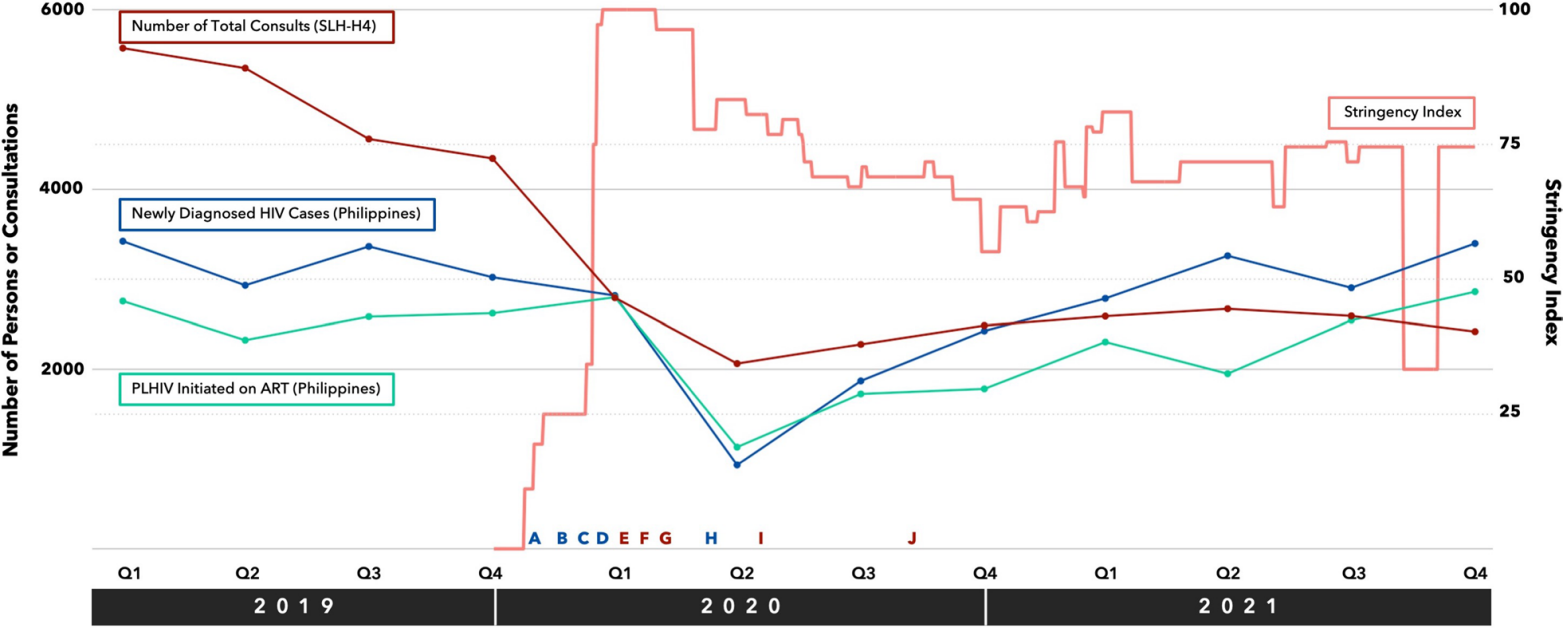
HIV care and COVID-19 in the Philippines and in San Lazaro Hospital. Red line indicates the number of total consults including new and follow-up clients at SLH-H4. Blue line indicates the number of newly diagnosed HIV cases in the Philippines. Green line indicates the number of PLHIV initiated on ART. Letters in blue indicate key events relating to COVID-19. Letters in red indicate key activities relating to HIV care. **A** (30 Jan 2020)—the first reported case of COVID-19 in the Philippines was admitted in SLH. **B** (7 Mar 2020)—community transmission was detected in the Philippines. **C** (11 Mar 2020)—COVID-19 was declared a global pandemic. **D** (16 Mar 2020)—Luzon Island including the National Capital Region was placed under enhanced community quarantine. **E** (Late Mar 2020)—SLH-H4 began referral of clients to other treatment hubs. **F** (Apr 2020)—SLH-H4 initiated ART delivery to clients using courier services. Fees were shouldered by clients. **G** (23 April 2020)—PhilHealth included the cost of parcel services under the outpatient HIV/AIDS treatment package. **H** (1 Jun 2020)—easing of restrictions began. **I** (Jul 2020)—The Department of Health adapted the use of parcel services for ART delivery in treatment hubs nationwide. **J** (Oct 2020)—SLH-H4 conducted a series of online lectures and fora for clients on key issues such as mental health, COVID-19, and nutrition

In the initial phase of the COVID-19 pandemic, the Philippines was placed in a state of public health emergency on 8 March 2020 [1], prompting a series of lockdown measures beginning in the National Capital Region (NCR) and further expanding to the rest of the country [2]. Containment measures for COVID-19 significantly impacted HIV programming in many contexts worldwide. In Japan, the number of HIV tests decreased by 50% in 2021 compared to previous years [3]. A time-series analysis of HIV care in South African primary care clinics found that lockdown was associated with a 48% reduction in HIV testing and 46% reduction in ART initiation in early 2020 [4]. In the Philippines, there was a 68% reduction in number of newly diagnosed HIV cases and 51.2% reduction in ART initiations during the second quarter of 2020 compared to the same period in 2019 [5]. Similarly, there was a 61.4% reduction in the number of consultations and 48.4% reduction in new patient enrollment during the second quarter of 2020 compared to the same period in 2019 in the SLH-H4 OPD (Fig. 1). In this letter, we share the experiences encountered and insights gained by the SLH-H4 department, an outpatient HIV health facility in a resource-limited setting, within the context of a challenged health system amidst the emergence of the COVID-19 pandemic.

## Ensuring uninterrupted access to life-saving health services

The imposition of enhanced community quarantine (ECQ) [6], the most stringent community lockdown in the Philippines, greatly affected HIV/AIDS services. Although the SLH-H4 OPD remained open during these times, the ban on use of public transportation, disproportionately affecting socioeconomically deprived communities, prevented individuals from accessing testing hubs and PLHIV from attending follow-up visits. To ensure uninterrupted access to vital services, the SLH-H4 team expediently coordinated referrals and temporary transfer of clients to nearby treatment hubs. Staff and case managers facilitated endorsements to hubs nationwide. In return, SLH-H4 accommodated transient services and medicine refills for clients not enrolled in, but had easier access to, the facility. However, some clients still felt hesitant to access hubs for fear of exposure to COVID-19.

By April 2020, the SLH-H4 utilized largescale courier services for delivery of ARTs to clients. The coverage of the courier services was initially limited within the NCR, but this was eventually expanded nationwide, spanning the three major islands of the country. This allowed continuous access to life-saving medications, subject to remote consultations with respective case managers and clinicians. The delivery fees were initially shouldered by clients. Partly due to the positive uptake and the practicality of this intervention, the National Insurance Agency (PhilHealth) included courier service charges into the outpatient HIV/AIDS treatment package by the end of the month [7]. This innovative undertaking was later integrated into national policies for wider use [8]. At present, the parcel service has been expanded to include diagnostic request forms for telemonitoring.

## Continuity of client education, empowerment, and social connection

As the pandemic persisted, other programs were affected. Central to the ethos of SLH-H4 was client empowerment facilitated through in-person learning group sessions (LGS) and peer-to-peer counselling. The SLH-H4 staff and case managers bridged the need for continued engagement via social media and online messaging apps. They quickly addressed the concerns on the risks of COVID-19 transmission and, later on, the challenges in securing vaccination appointments. Webinars on key topics such as mental health, nutrition, hygiene, and COVID-19 were conducted to further support the PLHIV community. The SLH-H4 team adapted to the rapidly changing lockdown policies to deliver LGS and counselling services safely, with positive response from attendees. For instance, the World AIDS Day 2020 celebration brought to SLH-H4 an unprecedented number of PLHIV participants who were requesting more in-person activities.

## Leveraging public–private partnerships

The COVID-19 crisis was also an opportunity for SLH-H4 to strengthen existing public–private partnerships (PPPs). Their non-governmental organization partners, namely AIDS Healthcare Foundation (AHF), Pharmaceuticals and Healthcare Association of the Philippines Foundations Inc. (PHAP-Cares), and Precious Jewels Ministries, played vital roles in supporting the unit’s innovative approaches. AHF facilitated procurement of drugs targeted for opportunistic infections, provided financial support in the delivery of online webinars and World AIDS Day, and helped augment SLH-H4’s manpower by providing two case managers. PHAP-Cares donated personal protective equipment (PPE) for healthcare workers in SLH-H4, which were also shared to the rest of the hospital workforce. Precious Jewels Ministries coordinated and shouldered transient services and ART refills for pediatric PLHIVs.

## Current challenges

Despite the great efforts of SLH-H4 to mitigate the impact of COVID-19 on equitable HIV/AIDS service delivery, challenges remain. At varying times during the pandemic, SLH-H4 staff contracted COVID-19 infection, forcing temporary clinic closures and limiting service provision. Additional support is necessary to protect the staff taking care of immunologically vulnerable populations. Some clients using the courier service relied heavily on remote ART refills, preventing regular clinical monitoring. Telemedicine may be utilized to bridge this gap, but establishing the platform is limited by poor internet connectivity in many areas, lack of dedicated human resources, and lack of data privacy and protection laws.

## Moving forward

Our experiences highlight the need to incorporate health service planning into pandemic preparedness policies. Risk assessment and mitigation measures from the national to the health-facility level should be strictly implemented [9]. The SLH-H4 staff and case managers play a key role in bridging care, and investments should be made to increase support for human resources for health. Regulatory and physical infrastructure for HIV telemedicine must be pursued continuously. Pooling of resources through functional PPPs to enable timely implementation of responses to challenges such as that posed by COVID-19 must be continually tapped. Finally, continued engagement with stakeholders, especially PLHIV, should guide HIV programming to achieve the 95-95-95 targets [10].

## Data Availability

Not applicable.

## Abbreviations

AHF: AIDS Healthcare Foundation
ART: Antiretroviral therapy
ECQ: Enhanced community quarantine
LGS: Learning group session
NCR: National Capital Region
PHAP-Cares: Pharmaceuticals and Healthcare Association of the Philippines Foundations Inc.
PLHIV: People living with HIV
PPP: Public–private partnerships
SLH-H4: HIV and AIDS Department, San Lazaro Hospital
